# Phenomenology of gender dysphoria in autism: a multiperspective qualitative analysis

**DOI:** 10.1111/jcpp.13691

**Published:** 2022-09-12

**Authors:** Kate Cooper, William Mandy, Catherine Butler, Ailsa Russell

**Affiliations:** ^1^ Department of Psychology, Centre for Applied Autism Research University of Bath Bath UK; ^2^ Research Department of Clinical, Educational, and Health Psychology London UK; ^3^ Department of Psychology University of Exeter Exeter UK

**Keywords:** Autism spectrum disorders, gender identity, gender dysphoria, adolescence

## Abstract

**Background:**

Autistic people are overrepresented in gender clinic settings, but limited evidence is available to guide clinical decision making for this patient group. We aimed to generate a comprehensive understanding of the phenomenology of gender dysphoria in autistic people.

**Methods:**

We conducted a multi‐perspectival interpretative phenomenological analysis (IPA), from five different perspectives; autistic young people and adults with experience of gender dysphoria, parents of young people, and clinicians working with autistic people with gender dysphoria in both adult and young person settings (*n* = 68).

**Results:**

IPA analysis resulted in two themes, ‘discovering gender identity’ and ‘the complexities of moving towards gender comfort’. Participants agreed that there was often an interaction between gender dysphoria and features of autism such as sensory sensitivities. There was relative consensus across groups about the need for autism adaptations to be made in gender clinics. Autistic adults were more likely to see autism as an important identity than young people, but both groups were clear that autism did not impair their understanding of gender. In contrast, some parents and clinicians working with young people expressed concern that autism did impact self‐understanding.

**Discussion:**

While the groups tended to agree on the ways in which particular features of autism can compound gender dysphoria, there were a range of perspectives on the ways in which autism impacted on self‐knowledge.

**Conclusion:**

Recommendations for adaptations when working with autistic people with gender dysphoria are presented.

## Introduction

Gender diversity is an umbrella term which describes individuals with gender identities or gender expression which do not conform to societal norms. This label includes those who are questioning or exploring their gender identity and those who are gender nonconforming, as well as those who identify as nonbinary and transgender. The term transgender refers to individuals whose gender identity does not match their assigned gender at birth. Individuals who are transgender or gender diverse may experience gender dysphoria, which is defined in the Diagnostic and Statistical Manual of Mental Disorders as distress due to incongruence between an individual's assigned gender and gender identity (5th ed.; DSM‐5; American Psychiatric Association, 2013). In the ICD‐11, the equivalent diagnosis is gender incongruence, and experiencing distress is not a criterion. There are debates about whether diagnostic criteria should exist in relation to gender incongruence, and if such criteria do exist, whether distress should remain central to the diagnosis (Beek, Cohen‐Kettenis, & Kreukels, 2016). Some individuals experiencing gender dysphoria seek assessment and treatment at gender clinics in order to explore their gender identity and expression and/or to access medical interventions to modify their bodies so they are aligned with their gender identity.

Autism is a neurodevelopmental condition characterised by atypical social communication and social interaction, as well as a restricted, repetitive pattern of behaviours, interests and activities (American Psychiatric Association, 2013). Rates of autism diagnoses in those attending gender clinics have been found to be between 5% and 26% (Cheung et al., 2018; Kaltiala‐Heino, Sumia, Työläjärvi, & Lindberg, 2015), compared with 1% in the general population (Brugha et al., 2016), and so autistic people are over‐represented in gender clinics.

The prevalence of mental health problems is elevated in both individuals diagnosed as autistic (Lai et al., 2019) and young people (YP) and adults who identify as transgender (Becerra‐Culqui et al., 2018; Nobili, Glazebrook, & Arcelus, 2018). Furthermore, adolescents and adults who are transgender or gender diverse and autistic are at yet higher risk of mental health problems (George & Stokes, 2018; Strang et al., 2021; van der Miesen, Hurley, Bal, & de Vries, 2018). Moreover, rates of suicidality are higher in both groups compared with the general population (Adams, Hitomi, & Moody, 2017; Hirvikoski et al., 2016). Autistic people experiencing gender dysphoria therefore represent a complex patient group; however, there is limited evidence available about how best to support people with co‐occurring autism and gender dysphoria. There are a number of cross‐sectional studies indicating the high co‐occurrence of being transgender or gender nonconforming and having an autism diagnosis (Warrier et al., 2020), and investigating the relationship between various autistic traits and diagnoses and being transgender or accessing gender clinics across the lifespan (for reviews see: Glidden, Bouman, Jones, & Arcelus, 2016; Øien, Cicchetti, & Nordahl‐Hansen, 2018; Thrower, Bretherton, Pang, Zajac, & Cheung, 2020; Van Der Miesen, Hurley, & De Vries, 2016).

Qualitative evidence about gender dysphoria in autism has generated some findings which are consistent with general accounts of gender dysphoria, with other findings suggesting that being autistic can contribute in particular ways to the experience. Strang et al. (2018) aimed to understand autistic and gender‐diverse YP's experiences in relation to gender and autism. Interviews with 22 gender‐diverse adolescents with verified autism diagnoses were analysed using framework analysis. Themes included participants' urgent needs to live as their affirmed gender and to make a medical transition, and confidence that their gender identity would be stable in future. Another theme was the impact of neurodiversity; for example, participants described difficulties in advocating for gender‐related needs.

Coleman‐Smith, Smith, Milne, and Thompson (2020) conducted a grounded theory study of the experiences of 10 adults with autism and gender dysphoria diagnoses in the United Kingdom. One overarching theme was identified: ‘conflict versus congruence’, referring to conflict between gender identity and body, conflict with other people, and psychological conflict. Some participants felt that autism was a barrier to making sense of their gender identity, and that the social environment prevented understanding and expression of gender identities. Making a gender transition was described as challenging due to social barriers including the social complexities of making a gender transition.

In work linked to the current study, we investigated the lived experience of gender dysphoria from the perspective of autistic adults (Cooper, Mandy, Butler, & Russell, 2021), YP and their parents (Cooper, Butler, et al., 2022), and clinicians working with autistic and gender‐diverse patients (Cooper, Mandy, et al., 2022). Autistic adults described experiencing distress due to a mismatch between their gender identities and bodies and identified ways in which autism intersected with gender dysphoria (Cooper et al., 2021). For example, participants experienced distress due to difficult social experiences, linked to both gender‐ and neuro‐divergence. The results of interviews with autistic YP (Cooper, Butler, et al., 2022) indicated a tendency to be focused on alleviating gender dysphoria through physical interventions, which could only be accessed through support from adults. Parents tended to be more focused on their children's needs linked to autism than gender. Clinicians identified some ways in which autism intersected with gender from their perspective, including through sensory needs, and different thinking styles. Clinicians also identified challenges for autistic people accessing gender clinics such as communicating their needs with clinic staff (Cooper, Mandy, et al., 2022).

While there are no treatment‐focused studies investigating the efficacy of gender‐affirming treatments such as cross‐sex hormones in autistic people, one novel study has aimed to design a clinical programme for transgender autistic adolescents (Strang et al., 2020). Interviews were conducted with autistic and gender‐diverse YP, advocates, parents and expert clinical providers. Themes included the need to provide clinical support for broader needs than those linked to gender, including social and executive function differences, and that parents needed support as well as their children.

Taken together, these findings support the hypothesis that an understanding of the autistic experience may be essential in effectively supporting needs related to gender dysphoria. However, debate in this field is characterised by polarised positions (Burki, 2019; Pearce, Erikainen, & Vincent, 2020), depending on the stakeholders' perspectives and experiences, and so it is essential to account for multiple perspectives when researching this area. More research is needed to understand similarities and differences in viewpoints on the intersection of gender dysphoria and autism across multiple groups, including autistic individuals, parents and clinicians, building on previous important work (e.g. Strang et al., 2019; Strang et al., 2020). Furthermore, research has often been designed from an outsider perspective by autism professionals and academics, but there have been calls to centre the voices of autistic and transgender people, with qualitative research being an important method to redress the balance (Strang et al., 2019; Strang, Knauss, et al., 2020).

According to a government investigation (Westminster Commission on Autism, 2016), healthcare provision for autistic people is inadequate, with 74% individuals from the UK autism community (*n* = 497) reporting that autistic people receive ‘worse’ or ‘much worse’ health care than nonautistic people. More research is needed to understand the adaptations needed in healthcare settings to better meet the needs of autistic people with gender dysphoria. Autism is a developmental condition, so to fully understand any intersection between autism and gender dysphoria, the experience of both autistic YP and adults should be ascertained.

This study aims to generate robust findings about the phenomenology of gender dysphoria in autism by identifying converging and diverging viewpoints on this intersection. We use a multiperspective qualitative synthesis design to compare the perspectives of autistic YP and adults with experience of gender dysphoria, parents of the YP and clinicians with experience of working with this patient group across the lifespan.

## Method

### Study design

We used multiperspective interpretative phenomenological analysis (IPA; Larkin, Shaw, & Flowers, 2019) to investigate the experiences of autistic YP and adults with gender dysphoria. A patient and public involvement group provided advice on the research question and design of this study (see Appendix S1). Autistic transgender adults and YP, their parents/carers and clinicians were all consulted. They contributed to co‐design of the research question, study materials including the information sheet and topic guide, and dissemination plan.

### Participants

Participants were autistic adults who had experienced gender dysphoria^1^ (*n* = 21), autistic YP who had experienced gender dysphoria (*n* = 15), parents of autistic YP (*n* = 16) and clinicians who work closely with autistic people with gender dysphoria, in adult settings (*n* = 8), and young person settings (*n* = 8). Recruitment was conducted between December 2019 and May 2021. See Table 1 for demographic information about the autistic participants, and Tables S1 and S2 for demographic information about all other groups. Young people and parents were predominantly recruited as parent–child dyads (*n* = 28), but one individual young person and two individual parents took part where their family member declined to participate. Participants were recruited from NHS gender clinics, mental health and autism services, community Lesbian, Gay, Bisexual, Transgender & Queer/Questioning (LGBTQ) groups and through contacts of previous participants. We used purposive sampling, so that autistic participants were varied in terms of geographical location within the United Kingdom, gender identity and stage of gender journey. Our approach was to simultaneously target a range of settings to access participants with different characteristics, for example gender and autism clinics in different geographical regions and LGBTQ community groups, which were likely to have members in the early stage of their transgender journey. Inclusion criteria for autistic participants were formal diagnosis of autism from a healthcare professional and having experienced gender dysphoria to the extent that they had sought professional support. Furthermore, participants were only included where they had the verbal and literacy abilities to be able to read and understand the information sheet and questionnaires for the study, and so were highly likely to have intellectual abilities within the normal range.

**Table 1:** Autistic participants

	Adults		Young people	
	*n*	%	*n*	%
Gender				
Male	7	33	9	60
Female	8	38	3	20
Non‐binary/Genderqueer	6	29	3	20
Sex assigned at birth				
Male	9	43	3	20
Female	12	57	12	80
Sexual orientation^a^				
Straight	0	0	2	13
Lesbian or gay	10	47	2	13
Bisexual	3	14	6	40
Asexual	4	19	0	0
Other	4	19	5	33
Ethnicity				
White British	20	95	14	93
Mixed	1	5	1	7
Gender transition undertaken				
Process not started	4	19	1	7
On gender service waitlist	4	19	6	40
Assessment at gender service	1	5	2	13
Hormones prescribed^b^	4	19	6^b^	40
Surgery – ongoing	6	29	–	–
Physical transition complete	2	10	–	–

	Mean	*SD*	Range	Mean	*SD*	Range
Age	29.1	11.5	18–51	15.7	1.28	13–17
Age in years of autism diagnosis^a^	22.45	13.62	3–51	12.50	3.80	3–17
Age in years realised transgender/non‐binary^a^	10.24	5.10	0–19	9.73	2.92	4–16
Age in years came out as transgender/non‐binary^a^	20.74	8.30	14–47	12.67	3.83	0–16
Age in years requested professional help for gender dysphoria^a^	22.16	7.99	16–47	13.29	2.09	8–16
Time from realisation to disclosure of gender identity	10.16	11.20	1–41	3.20	2.40	0–9
Time from realisation to requesting support	11.37	11.03	1–41	3.57	2.24	0–9

^a^ As described/reported by participants.

^b^ Half of these were prescribed privately, half in the NHS, includes puberty blockers.

Gender dysphoria diagnosis was not an inclusion criterion due to the rapidly shifting definitions within the field of transgender health (Beek et al., 2016), because autistic people may experience gender dysphoria in different ways to the broader population and to capture individuals at different stages of their gender journey. Young people were aged between 13 and 17 years, and adults were 18 years or older. Clinicians needed to have experience working with either group in a healthcare setting.

### Procedures

All participants were required to share key demographic details, and autistic participants were asked to complete questionnaires. The Gender identity/gender dysphoria questionnaire for adolescents and adults was used to measure gender dysphoria and is validated for use in adolescents and adults (Deogracias et al., 2007), with 27 items such as ‘In the past 12 months, have you felt satisfied being a girl/boy/woman/man?’, with each item scored between 1 (‘always’) and 5 (‘never’), with lower scores indicating higher gender dysphoria. The Patient Health Questionnaire‐9, a widely used and well‐validated measure of depression in adults (Kroenke, Spitzer, & Williams, 2001) and adolescents (Richardson et al., 2010), has 9 items including ‘Over the past 2 weeks, how often have you been bothered by any of the following problems: Little interest or pleasure in doing things’, which are scored from 0 (‘not at all’) to 3 (‘nearly every day’), with higher scores indicating higher levels of depression. Suicidal behaviours were measured using the four‐item Suicidal Behaviours Questionnaire‐Revised, validated for use in adolescents and adults (Osman et al., 2001). Each item has different response options, for example ‘how likely is it that you will attempt suicide someday?’ and has responses from 0 (‘never’) to 6 (‘very likely’), with higher overall scores indicating higher risk of suicide. Finally, psychological well‐being was measured using the Short Warwick–Edinburgh Mental Wellbeing Scale, validated for use in adults (Fat, Scholes, Boniface, Mindell, & Stewart‐Brown, 2017) and adolescents (Clarke et al., 2011), which has seven items such as ‘I’ve been feeling useful’, all scored from 1 (‘none of the time’) to 5 (‘all of the time’), and higher scores indicate better psychological well‐being.

The first author interviewed all participants using a flexible topic guide, adapted for autistic interviewees, and based on IPA guidelines (see Appendix S2). Topics included the phenomenology of gender dysphoria in autistic individuals; interaction between autism and gender dysphoria; experiences in gender and mental health clinic settings. For example, one item for autistic participants was ‘Do you feel that being autistic has affected your personal experience of gender dysphoria?’ Autism adaptations were made to improve communication between the non‐autistic interviewer and autistic participants. Social communication adaptations included modifications to interviewing style, for example the interviewer used closed questions when needed; used multiple modes of communication, for example voice calls, video calls and synchronous text messaging; and there was the option to have a carer present during the interview to support communication. With respect to the restricted and repetitive behaviours domain of autism, adaptations included refocusing participants who started to talk at length about other topics such as special interests; ensuring the sensory environment was comfortable for the participant by, for example turning lights off or opening a window; and encouraging access to fidget toys where the participant had brought these to the interview. Interviews were audio recorded by the first author and professionally transcribed by an external transcription company, with transcriptions then checked by the first author to ensure their accuracy.

### Ethics considerations

The study received ethics approval from the Health Research Authority (19/NE/0265). Recruitment took place before and during the COVID‐19 pandemic, and study participation was in‐person or remote via a video or phone call. Fully informed consent was gained from all participants (or assent and consent from parents for participants below the age of 16 years), and ethical procedures were followed throughout.

### Data analysis

The questionnaires were administered to describe the sample and are presented in Table 2. Qualitative analysis followed the guidance of Larkin et al. (2019) for a multiperspectival IPA study (see Appendix S3 for further detail on the analytic approach) and was conducted concurrently with data collection. The first author analysed each individual transcript (*n* = 68), noting the descriptive, linguistic and conceptual elements, focused on the meaning of the intersection of autism and gender dysphoria to participants. Next, important themes were generated for each participant focused on when the notes had similar or oppositional ideas or functions within the interview, and considering important contextual factors. Then, we drew together themes for each unit of study (adults; YP and parent dyads, and then the whole group of YP and parents; adult clinicians; young person clinicians, and then both clinician groups), and analyses at this level have been published elsewhere (Cooper et al., 2021; Cooper, Butler, et al., 2022; Cooper, Mandy, et al., 2022). Themes were included when they were endorsed by at least 50% of participants, following recommendations for high‐quality IPA research with larger samples (Smith, Flowers, & Larkin, 2009). Quotes were selected when they were a good summary of the core of a theme and to present views across a wide range of participants. We followed guidance for quality IPA analysis (Nizza, Farr, & Smith, 2021) and practised reflexivity and quality checks throughout (see Appendix S4). These included the first author presenting the analysis in supervision, including IPA supervision groups and the patient and public Involvement group to ensure a rigorous and credible analysis was conducted.

**Table 2:** Gender dysphoria and mental health measures

		Adults (*n* = 21)			Young people (*n* = 15)		
Measure	Cronbach's alpha^a^	Mean	*SD*	*n* (%) with clinically significant score^b^	Mean	*SD*	*n* (%) with clinically significant score
Gender dysphoria (GIDYQ_AA^c^)	50–84	2.26	0.46	19 (91%)	2.18	0.38	14 (100%)^d^
Depression (PHQ9^e^)	88–90	13.29	6.54	13 (62%)	16.60	6.91	13 (87%)
Suicidal Behaviours (SBQ‐R^f^)	78–80	10.26	4.56	11 (58%)	12.00	4.07	13 (87%)
Well‐being (WEMWBS^g^)	57–83	20.05	3.46	7 (33%)	19.53	5.17	10 (67%)

^a^ Calculated for each measure separately by participant group (adults and young people).

^b^ Defined as <3 on the GIDYQ‐AA, ≥10 on the PHQ‐9, >8 on the SBQ‐R, <19 on the WEMWBS.

^c^ GIDYQ‐AA = Gender Identity and Dysphoria Questionnaire‐Adolescents and Adults; Scores <3 indicate gender dysphoria.

^d^ One participant declined to complete this measure.

^e^ PHQ9 = Patient Health Questionnaire; ≥10 indicates moderate depression; ≥15 indicates moderately severe depression.

^f^ SBQ‐R = Suicide Behaviours Questionnaire‐Revised; >8 indicates high risk in psychiatric inpatient samples.

^g^ Warwick–Edinburgh Well‐being Scale; scores <19.6 indicate significantly low well‐being.

This study represents a new, synthesised analysis, which aims to understand the convergence and divergence in views across the five participant groups, generating unique and robust findings beyond those already published. It was necessary to report individual group findings separately due to the high number of participants recruited for an IPA study, depth of analysis conducted and need to represent individual participant perspectives in detail at each level of the analysis, and because the current analysis has resulted in new findings which were only evident through the synthesis of the five different participant groups. Focusing the analysis on the intersection of autism and gender dysphoria from a range of perspectives deepens the overall understanding of this phenomenon and provides an account of the similarities and differences in perspectives between five different groups, which has not been possible in previous publications (see Appendix S3). There is a precedent for publishing multiple studies from IPA analyses (Osborn & Smith, 2006, 2008), and this method follows a long history of qualitative research synthesis (Larkin et al., 2019; Noblit & Hare, 1988).

## Results

The demographic questionnaires (Table 1) indicated that, on average, both adult and adolescent participants realised that they were transgender at 10 years old, preceding the average age of autism diagnosis. Autism diagnoses were at the average age of 13 and 22 for adolescents and adults respectively, with 48% of adult participants receiving an autism diagnosis in childhood. Both autistic adults and YP had GIDYQ‐AA scores indicating clinically significant gender dysphoria. Autistic adults had depression scores in the moderate range (≥10), while YP had moderately severe depression scores (≥15). Both groups had well‐being scores indicating possible anxiety or depression, and suicidal behaviour scores indicating high risk of suicide.

The novel multiperspective synthesis resulted in two overarching themes, each with two subthemes, which were (1) discovering gender identity and (2) the complexities of moving towards gender comfort. See Table 3 for a key for participant ID codes.

**Table 3:** Participant IDs

Participant group name	Description	Participant ID
Adult participants	Autistic adults with experience of gender dysphoria	e.g. A1
YP participants	Autistic YP aged 13–17 with experience of gender dysphoria	e.g. YP1^a^
Parent participants	Parents of the YP	e.g. P1^b^
Adult clinicians	Clinicians who work with autistic adults with experience of gender dysphoria	e.g. AC1
YP clinicians	Clinicians who work with autistic YP with experience of gender dysphoria	e.g. YPC1

^a^ Same number for young person and parent indicates parent–child relationship.

^b^ As described/reported by participants.

### Discovering gender identity

Gender was conceptualised as an important identity, which needed to be discovered by the individual, their family and clinicians. Autism was seen as influencing that discovery, by impacting on one's sense of self. Autistic adults considered their autism identities as more central to their sense of self as compared to autistic YP. From the perspective of some parents, autism caused difficulties, which were more significant than those arising from their child's gender identity and associated dysphoria.

#### Gender as a deeply known and central identity

Gender identity was described as an immutable and important part of the self, with knowledge of this gender identity generally described as unfolding over time. Autistic participants described the depth and length of their experiences of their gender identities, as evidence of their veracity, for example a transgender male participant said that his gender identity was ‘very deep, very young’ (A1). Autistic participants in both age groups described negative experiences of their bodies being at odds with their identities, for example YP2 described feeling ‘quite disgusted with myself in a way … I always think that it’d be a lot easier if I was a female on the inside and the out instead of the boy inside and then the female out.’

Alongside this understanding of gender identity as knowable was the distress felt by participants when experiencing uncertainty about gender. Participant A23 grappled with this: ‘…for me in everything I do, in everything I think, there has to be some sort of like definitive truth to everything and so like I said before I had a doctor who said, ‘do you feel like a woman?’, well I don’t know what a woman feels like because I mean I wasn’t born one… I’m not happy with saying yes I feel like a woman when I don’t have every available fact’.

Parents of autistic YP tended to agree that gender identity should be certain and known, particularly before physical treatments were accessed which could change the body, such as P9: ‘I think she just doesn’t know where she is and she’s trying to find someone that she can identify with and I’m guessing she’s finding it. I don’t know. She seems more certain on it than I do. It’s like I’m seeing different.’ YP clinicians corroborated this need for certainty with their clinical aims to go ‘below’ (YPC11) and deepen their understanding of their patients' gender identities in order to ensure that YP and parents were making the right decisions.

#### The role of autism in developing self‐understanding

Adults often considered autism as an important part of themselves, describing autism diagnosis as increasing self‐understanding, such as A17: ‘after having a diagnosis [of autism] a lot more of my experiences have come to light’. In contrast, when YP participants were asked about autism, their responses were frequently minimal and detached, with more of a tendency to separate themselves from autism, such as YP5: ‘It’s not like I’m walking autism and that’s all there is to me.’

Adults described autism as interacting with their gender identities, but not having a negative impact on self‐understanding, such as A3 who described the relationship between autism and understanding gender identity: ‘I think there are intersections, but I don’t think it [autism] completely jades my perception.’ Young people often distinguished their autism and gender identities, seemingly in order to demonstrate the reality of their gender identity: ‘And maybe I think because I’m autistic I’m very good at sort of piecing together facts and getting information and applying it to something else which I think I probably did with my gender, but I think I would also definitely feel this way if I wasn’t autistic’ (YP8).

While autistic participants did not describe autism as a causal factor in their gender identities, many parents and YP clinicians felt that autism played an important role and that autistic YP needed to consider their autism to fully understand their gender identity. In contrast to the YP, parents often saw their child's autism as a more influential part of their child than gender, such as P8: ‘the biggest factor is there are other issues and the sensitivities they have around their autism, which I think swamps a lot of other things.’ An underlying fear was that YP might make a decision to physically transition which they would later regret, without full understanding of the role of autism, for example YPC16 said: ‘I felt my work was often about wanting to help them have a kind of richer sense of their gender experience to help them link it with other aspects of their lives, like their autism. But particularly where young people were wanting medical intervention, I think they found that quite hard to tolerate … as though acknowledging those aspects might make it less likely they’d get the medical treatment.’

Adult clinicians wanted to understand their patients and aimed to discover how gender and autism related to one another for the individual, such as AC3: ‘I think having autism can affect your experience of yourself particularly yourself in relation to others and that’s quite crucial in how we all relate to identity …It could also introduce more uncertainty.’ Autistic adults often described, and YP sometimes described the ways that autism could influence the process of coming to know one's gender identity. For example, participant A9 said that their style of thinking linked to autism had slowed down their process of discovery: ‘I feel like if I didn’t have the autism that I have and I was able to be more critical and analytical, then I might have been able to come to a conclusion on my true gender identity earlier than I did.’ In contrast, some YP and adults made it clear that autism had helped them to work out their gender identity.

Some parents and YP clinicians were concerned that difficulties in social interaction and social understanding might contribute to difficulties understanding oneself, such as P11: ‘It’s very difficult because as a parent, I can’t definitely say whether [child] is transgender, lesbian, or bisexual. All I know is that he has got autism, he does tend to sometimes take on influences of his peer group, and it’s really difficult.’ This was a fear which YP themselves dismissed, in the words of one young person: ‘I know this is who I am.’ (YP11).

### The complexities of moving towards gender comfort

The second superordinate theme describes how autistic participants wanted to move away from distress and towards gender comfort, but that autism could sometimes intensify gender dysphoria. There was relative consensus across groups on how autism features tended to interact with gender dysphoria in autistic people. Most autistic participants wished to undertake a physical gender transition at a gender clinic, to alleviate distress, but there were sometimes barriers to accessing this as an autistic person.

#### Autism compounding gender dysphoria

Many autistic participants, parents and clinicians described an interaction between autism sensory needs and negative experiences of the body, which intensified gender dysphoria. One adult (A18) described this interaction: ‘I was stuck between having really bad gender dysphoria not wearing a binder or feeling really uncomfortable sensory wise.’ Puberty often heightened these experiences of ‘sensory dysphoria’ (A1), such as for one young person who described his experience of puberty: ‘This is awful, I hate this, I want to stop this as soon as I can’ (YP7).

Clinicians and autistic participants also described how difficulties managing change could contribute to distress when making a gender transition, for example YP10 said: ‘I feel like as I do more medical things that change like my body, the routine of that is gonna be very, very hard.’ One parent described how puberty represented change that was out of their child's control, which was distressing due to both autism and gender dysphoria: ‘I think with the fact that his body went from being similar to his brother’s to having a chest and having periods, I think has really thrown him. He’s no longer in control of his body’ (P15).

Autistic participants, parents and clinicians, described how rigid thinking associated with autism sometimes related to gender dysphoria, such as YPC16: ‘I think their autism impacts on their experience of gender [as] a real desire to systematise their gender experience and explore labels that are made to define and categorise … my sense was that it was part of their defence sometimes against the unknown nature of things, living, changing, evolving nature of being that person.’ Some autistic adults described fearing that their gender identity might be a special interest but concluding that this was not a helpful way of understanding their experiences, and clinicians tended to agree: ‘I suppose the worry was always that, within the autistic framework, that gender was seen like an obsession or a specialist interest. I never really saw it in that sense.’ (YPC8).

Participants also described how social differences could increase distress linked to gender dysphoria. An adult participant described how he struggled to navigate the social complexity of making a gender transition: ‘You change your name and suddenly you’re wearing the same things you’re the same person you look the same you sound the same … One day you’re referred to as female and Miss and expected to use the women’s toilet and the next day it’s Mr and you probably should be shot for going into a women’s toilet. It’s like that whole set of rules is really, really strange and most things that I do are based on like algorithm flow charts’. (A16).

#### Clinical services perceived as a barrier

Clinical services were perceived as a barrier to gender comfort, as the majority of autistic participants who wished to make a physical transition felt that gender clinics could provide the support they needed, but good communication and autism adaptations were sometimes lacking during assessments. Autistic YP and adults sometimes struggled to understand healthcare clinicians, while at times, clinicians struggled to understand autistic patients. This led to frustration on both sides. For example, participant A13 said:

I feel like my autism kind of gets ignored in a way. Like I have the diagnosis. It’s on my file, but no one really bothers. No one’s really acted any kind of differently towards it. With my autism I need kind of things explained and stuff like that. You can’t talk to me very professionally, otherwise I won’t understand and that kind of stuff. They just make no kind of alterations at all.

An adult clinician elaborated:

you’re taught an awful lot of sort of reflective interviewing and checking whether the other person’s got it and asking them how they feel and this sort of stuff, all of which deeply disturbs … autistic people… The hard thing actually clinically is shifting gear between neurotypical and non‐neurotypical as one patient succeeds another. (AC6).

Participants described attempts to alleviate the frustration felt by both groups in appointments where there was a mismatch of communication style. Autistic participants and clinicians alike described a similar set of adaptations that made, or would have made, appointments more comfortable and accessible for autistic patients. These included changes to the structure of appointments, clinic environments and clinician communication style. There was relative consensus about the need for autism adaptations to be made in gender clinics, and suggestions for these, based on the noting stage of the analysis, can be found in Table 4. These were grouped into three overarching categories: (a) structure of appointments including location and clinical tasks, (b) environmental adaptations including clinic setting and sensory considerations and (c) communication adaptations focused on ensuring accessible communication by clinicians.

**Table 4:** Summary of suggested adaptations for autistic people seeking support for gender dysphoria

Structure of appointments
Having proper notice for appointments
Support with long journeys to clinics or option for remote appointments
Provide a clear outline of a typical process of attending a gender clinic and different gender journeys
Have an initial ‘say hello’ appointment with no specific clinical tasks
Have a parent, partner or other supporter at appointments
Have a regular clinician for each appointment
Have longer or shorter appointments as needed
Order the agenda of appointments considering patient energy and comfort levels
Environmental adaptations
Ensure gender neutral toilets are available
Demonstrate LGBT and autism awareness in waiting areas and clinic
Clinician awareness of the sensory environment at clinics
Quiet waiting area
Consider turning off lights or loud equipment, for example clocks and computers
Consider temperature in clinics – consider patient control over opening windows etc
Communication adaptations
Check patient understands the purpose of questions asked in assessments
Use clear, slow, nonpatronising communication without use of technical language
Use forced‐choice questions or open questions with prompts
Present information in manageable chunks
Allow time for the patient to respond and check understanding
Minimise gesture, eye contact and use an even tone and volume when speaking
Provide support and structure for patient to identify and communicate about emotions
Use special interests to increase engagement
Use written and visual resources as needed, for example videos, charts, pros and cons lists, tables, diagrams and gender maps
Check preferences for remote communication, for example using emails rather than phone calls

## Discussion

Five different participant groups highlighted the ways in which autism and gender dysphoria intersect from multiple perspectives, with considerable convergence in viewpoints, particularly relating to how features of autism can compound gender dysphoria and in clinical barriers to getting support. This method has allowed for important new findings beyond those previously published (Cooper et al., 2021; Cooper, Butler, et al., 2022; Cooper, Mandy, et al., 2022), particularly in the ways in which autistic YP and adults converged and diverged in viewpoints, providing a developmental perspective on the relationship between autism and gender dysphoria, and in significant differences in opinion between clinicians, parents and autistic YP. These findings build on previous work by allowing a direct comparison between groups, generating new findings which are critical to clinical decision‐making for this group since conversations between clinicians, autistic people and in the case of YP, their parents, are central to the diagnosis and treatment of gender dysphoria in current medical practice.

There was generally agreement between participants that gender was a deep, immutable and knowable part of the self, and that a mismatch between gender identity and sex led to significant distress, in line with the diagnostic criteria for gender dysphoria (American Psychiatric Association, 2013). However, a novel finding in this study was significant divergence between groups when considering the role of autism in coming to know one’s gender identity, which suggest a developmental perspective is much needed. Autistic adults were more likely than YP to consider autism an important part of the self and were more willing to discuss autism and how it intersected with other parts of themselves, where autistic YP were less comfortable discussing autism and preferred to focus on their gender identity. Previous qualitative findings have indicated that autistic YP often distance themselves from the autism label (Calzada, Pistrang, & Mandy, 2012; MacLeod, Lewis, & Robertson, 2013). Recent quantitative research demonstrated that length of time since autism diagnosis was associated with more positive well‐being: it is possible that over time, autism diagnoses are assimilated into one's sense of self‐leading to more positive outcomes (Oredipe et al., 2022), and the qualitative findings in this study support this hypothesis. Parents and YP clinicians often placed more emphasis on autism as an important part of their child's identity than the YP themselves. This finding may reflect the needs which are most salient from an external perspective, with gender needs more hidden than autism‐related needs (Legg & Tickle, 2019), and has overlaps with research with parents of autistic and gender‐diverse YP in the United States, 60% of whom stated that neurodiversity related needs could be more complex to navigate than gender‐related needs (Strang et al., 2021). However, in contrast to Strang, Powers, et al.'s (2018) finding that YP had the experience of their gender identity being dismissed as an obsession, participants in this study, including autistic individuals and clinicians, upon reflection did not consider gender diversity to be linked to special interests or obsessional thinking.

Autistic participants in both age groups were clear that autism did not negatively impact on their knowledge of their gender identity, although some autistic participants stated that autism could influence the discovery of this knowledge. Previous work with autistic transgender youth similarly found that autistic YP were confident that their gender identity would not change (Strang, Powers, et al., 2018). In contrast to autistic people themselves, YP clinicians and parents sometimes felt that autism did influence gender knowledge, particularly through social differences, which is concordant with previous findings that autistic YP felt their gender identities were likely to be questioned and undermined by others due to being autistic (Strang, Powers, et al., 2018). In contrast, clinicians working with adults more often focused on the intersection (rather than causal relationships) between features of autism and gender dysphoria. This difference between adult clinicians and parents and YP clinicians may be due to the developmental trajectories of gender identity development. There are no large‐scale longitudinal studies indicating gender identity trajectories for autistic YP throughout development, although of 22 autistic YP meeting criteria for Gender Dysphoria at the beginning of one longitudinal study, two identified as cisgender after 22 months (Strang, Powers, et al., 2018). Identity development is conceptualised as a task of adolescence, although there is limited evidence about gender identity developmental trajectories, and one recent study suggests that YP with features of gender dysphoria do not have impaired identity development (Karvonen, Goth, Eloranta, & Kaltiala, 2022). Nonetheless, ideas about developmental trajectories of identity development may influence differences in opinion between those supporting adults compared to YP, with more concerns from YP clinicians and parents that YP may have different identities by adulthood. Free from such developmental considerations, clinicians working with adults were able to focus more on the intersection of autism and gender without imperative to consider any causal relationships between the two.

Crucially, there was significant convergence in views on the ways in which being autistic may compound gender dysphoria, through the interaction of features of both conditions. These were the interaction between gender dysphoria and sensory sensitivities, resistance to change, rigid thinking and social differences. Special interests were not seen as contributing to gender dysphoria. Furthermore, there was relative consensus on adaptations which would improve the experience of autistic people with gender dysphoria when accessing health services, and these adaptations address barriers similar to those identified by autistic adults accessing mental health (Brede et al., 2022) and physical health services (Nicolaidis et al., 2015). There are overlaps in this theme with Strang, Powers, et al.’s (2018) finding that autism could impact gender discernment and communication about gender in autistic youth, and that parents also identified such challenges in clinical settings (Strang et al., 2021). There was evidence of the double empathy problem between clinicians and autistic patients, whereby communication difficulties are conceptualised as being caused by a reciprocal difficulty in autistic and nonautistic individuals understanding one other (Milton, 2012). Clinicians working with autistic people experiencing gender dysphoria should be offered training in adapting their practice for autistic individuals. Table 4 outlines specific ways in which this could be done, as suggested by all participant groups and noted during the IPA analysis. These include communication adaptations which may reduce the likelihood of miscommunication between autistic patients and clinicians, for example by clinicians using less nonverbal social communication such as gestures during appointments in order to reduce the social demand on the autistic individual. Other adaptations such as to the structure of appointments aim at increasing comfort, including having a regular clinician and control of the sensory environment, which in turn might improve communication and productivity in appointments. These recommendations focus on adaptations to clinic processes and communication, rather than to treatment decision‐making, since such recommendations should be based on longitudinal quantitative research. However, there are existing clinical guidelines linked to decision‐making for adolescents based on a Delphi study with experts, which recommends a gender and autism specialist team, evaluating for autism‐related differences that may affect communication, assessment of the role of autism in the development of gender dysphoria, and addressing both autism and gender‐related needs concurrently (Strang et al., 2018).

The autistic participants recruited in this research completed quantitative measures of their mental health and the average scores indicated clinically significant gender dysphoria, depression and suicide risk. This demonstrates that participants had significant mental health needs, in line with research indicating elevated risk of mental health problems in autistic and transgender individuals (Strauss et al., 2021). It should be noted that there was low internal consistency for the gender dysphoria and well‐being questionnaires in adults, so these quantitative findings should be interpreted with caution given the low alphas and small sample size. The quantitative results are consistent with the qualitative finding that the features of autism could compound gender dysphoria, increasing overall distress.

This study recruited a wide range of participants with different perspectives on the intersection of autism and gender dysphoria. A limitation is the lack of cultural diversity, which is reflective of autism and transgender health services (de Graaf, Manjra, Hames, & Zitz, 2019). Another limitation is comparing findings between autistic adults and YP who represent different demographic groups; YP were more likely to be assigned female at birth, in line with current referrals to YP's gender services in the United Kingdom (Morandini, Kelly, de Graaf, Carmichael, & Dar‐Nimrod, 2021). Therefore, these findings are likely to extend to autistic people accessing gender clinics, but the demographic features of this population have changed rapidly in recent years (Arnoldussen et al., 2020) and may continue to do so, and so considering contextual factors will be crucial in understanding these results in future. Furthermore, it was not possible to record characteristics of potentially eligible participants who declined to share their details with the research team: possible factors for declining to participate could be having less positive rapport with healthcare clinicians, less investment in one's autism diagnosis, more severe mental health needs or in the case of YP, having parents who are unsupportive regarding their gender identity: such individuals may therefore be underrepresented in our sample. Half the autistic adults were diagnosed with autism in adulthood and therefore may well have had different perspectives on autism (Oredipe et al., 2022). However, a strength of IPA analysis is that context and idiography directly inform the analysis, so these different perspectives and life experiences are considered throughout the analysis. Moreover, including a range of perspectives strengthens the convergent findings about the relationship between autism and gender dysphoria, shedding light on this increasingly common co‐occurrence.

## Supporting information


**Table S1.** Parent demographics.
**Table S2.** Clinician demographics.
**Appendix S1.** Patient and public involvement.
**Appendix S2.** Topic guide for each study.
**Appendix S3.** Analytic approach.
**Appendix S4.** Reflexivity statement.

## Note

1 In this paper, Gender Dysphoria refers to the DSM‐5 definition, while gender dysphoria written in the lower case refers to the experience of distress in relation to an incongruence between assigned gender and gender identity, in which case, an individual may or may not meet DSM‐5 criteria.
