# Supplementary material for: Phenomenology of gender dysphoria in autism: a multiperspective qualitative analysis
Source: J Child Psychol Psychiatry. 2022 Sep 12;64(2):265–76. doi: 10.1111/jcpp.13691 (PMC10087378; doi:10.1111/jcpp.13691)
Supplement: Supplementary file 1 — Table S1. Parent demographics. Table S2. Clinician demographics. Appendix S1. Patient and public involvement. Appendix S2. Topic guide for each study. Appendix S3. Analytic approach. Appendix S4. Reflexivity statement. [file JCPP-64-265-s001.docx]

**Supporting Information**

**Table S1**.

Parent demographics

| **Gender** | n | % |
| --- | --- | --- |
| *Male* | 2 | 13% |
| *Female* | 14 | 88% |
| **Sexuality** |  |  |
| *Straight* | 16 | 100% |
| **Ethnicity** |  |  |
| *White* | 15 | 94% |
| *Mixed* | 1 | 6% |
|  | **Mean** | **Range** |
| ***Age in years*** | 48 | 42-55 |

| Gender | **n** | **%** |
| --- | --- | --- |
| Male | 3 | 19% |
| Female | 13 | 81% |
| Professional background |  |  |
| Medical doctor (inc. Psychiatry) | 3 | 19% |
| Nurse (inc. mental health nurses) | 2 | 13% |
| Therapist | 1 | 6% |
| Social worker | 1 | 6% |
| Clinical Psychologist | 9 | 56% |
| Total number of autistic and transgender patients |  |  |
| 10-24 | 5 | 31% |
| 25-49 | 3 | 19% |
| 50-99 | 2 | 13% |
| 100-150 | 4 | 25% |
| 200+ | 2 | 13% |
|  | **Mean** | **Range** |
| Years qualified in profession | 15.13 | 2-31 |
| Years worked with autistic people | 14.69 | 4-29 |
| Years worked with transgender people | 11.34 | 3-35 |

**Table S2.**

Clinician demographics

**Appendix S1. Patient and Public Involvement**

Before applying for funding to do this work, a Patient and Public Involvement group helped to develop the research questions, meeting twice in 2017 and 2018 to refine the study procedures. Once funded, this group were regularly consulted about the conduct of the research. The group members were autistic adults who had experienced gender dysphoria, along with some of their carers, where appropriate. Autistic young people, their parents, and clinicians were also consulted on an ad hoc basis outside of the established group. The group endorsed the proposed research questions and made suggestions for the methods including focusing on the lived experiences of both adults and young people. The group also suggested having a wide recruitment strategy with participants varied in geographical location, and age of onset of gender dysphoria, gender identity, as well as stage of gender transition. The group gave advice on recruitment including suggesting community groups from which to recruit participants. The PPI group members helped to ensure that autism adaptations were made to interviews, for example, offering the chance to conduct interviews through instant messaging and sending interview questions in advance to participants. At the stage of writing up the findings from the research, the group gave advice on labelling the themes so that they were accessible to the autistic community. This led to changes in the theme names, but not content. The PPI group also helped in the development of accessible summaries for the autism community, and training materials for NHS clinicians, to ensure that the findings were disseminated beyond the academic community.

**Appendix S2. Topic Guide for each study**

2a): Autistic participants

| **What has been your experience of being autistic and having gender dysphoria?**   - Thoughts, feelings, sensations - What is it like living with distress or discomfort relating to your gender identity? - Is there anything else we should know about your experience of living with gender dysphoria as an autistic person? | **How has gender dysphoria developed and changed over time?**   - Earliest memories - When did you notice that your gender identity was different compared to other people? - Change in gender identity over time? - Do you remember when you first felt distress or discomfort about your gender identity? - Have these thoughts, feelings and sensations changed over time? |
| --- | --- |
| **Do autism and gender dysphoria affect one another?**   - When were you diagnosed as autistic? - Has being autistic affected the way you see gender? - Do you feel that being autistic has affected your personal experience of gender dysphoria? - Social communication - Routines or repetitive behaviours, interests, and activities - Sensory differences | **Interaction of gender dysphoria and mental health**   - Has your experience of gender dysphoria affected your mental health? - Have there been times when you have felt like hurting yourself because of gender dysphoria? |
| **Seeking Help for gender dysphoria**   - First time you asked for help around gender dysphoria? - How did you decide to ask for help? How long did it take - Which professionals have helped you with gender dysphoria? - What was your experience of navigating NHS services to get help for gender dysphoria? - Is there anything else we should know about your experience of accessing NHS services for gender dysphoria? | **Adaptations in services**   - Did you feel that your autism was understood in services? - Was gender dysphoria understood in services? - Was there anything that got in the way of you getting help for gender dysphoria? - Can you give examples of things that helped you get support for gender dysphoria? - Any adaptations that should have been made for you? - Do you think that being autistic made any difference to you getting support for gender dysphoria? Did anything get in the way of getting help? |

2b): Parents

| **What has been your child’s experience of being autistic and having gender dysphoria?**   - Did you ever notice your child becoming distressed about their gender identity? How did this distress manifest – what behaviours did they display? What would they say to you about this? - Did you notice your child’s gender identity and distress relating to this change over time? - What do you think it has been like for your child living with their gender dysphoria? Are you aware of the thoughts, feelings and sensations they experience in relation to their gender dysphoria? | **How has your child’s gender dysphoria developed and changed over time?**   - When did you first become aware that your child had differences in their gender identity? - What did you make of these differences at this early stage? - Did you notice your child’s gender identity change over time? - When did your child first talk to you about their gender identity? |
| --- | --- |
| **Do autism and gender dysphoria affect one another?**   - When was your child diagnosed as autistic – did this come before or after they asked for help about gender dysphoria? - Do you feel your child’s autism has affected their experience of gender dysphoria? - Do you feel that having differences in social communication has affected your child’s experience of gender dysphoria? - Do you feel that your child’s routines or repetitive behaviours, interests and activities have affected their experience of gender dysphoria? - Do you feel that any sensory differences have affected your child’s experience of gender dysphoria? | **Interaction of gender dysphoria and mental health**   - Do you think your child’s gender dysphoria has affected their mental health? - Has your child ever talked about or actually harmed themselves because of gender dysphoria? |
| **Seeking Help for gender dysphoria**   - How did you decide to ask for help from a professional about your child’s gender identity? - Who did you ask for help from? - How many different professionals have you met with for your child’s gender dysphoria, and from which services? - What was your experience of navigating NHS services to get help for gender dysphoria? - Did you feel that your child’s autism was understood in services? - Do you feel that any interaction between autism and gender dysphoria was understood in services? | **Adaptations in services**   - Was there anything that got in the way of you getting help for your child? *For example, professionals or family members not understanding your needs, not knowing where to get help from etc.* - Can you give examples of things that helped you get support for your child’s gender dysphoria? What helped the most? - Do you think that your child’s autism made any difference to you getting support for their gender dysphoria? Did anything get in the way of getting help? - Is there anything else we should know about your experience of having an autistic child with gender dysphoria? |

2c) Clinicians

| **Demographics**   - What is your gender? - How many years have you been working as a qualified NHS professional? - What is your professional background? - What is the highest qualification you have achieved, and what is the name of this qualification? - How many years have you worked with autistic people? - How many years have you worked with transgender people? - Approximately how many autistic transgender people have you worked with? - Have you experience working with autistic transgender people of a different age? (i.e. adults if in a child setting and vice versa) | **Do autism and gender dysphoria affect one another?**   - Do you think autistic people experience gender related distress differently to other people? If so, how? - Do you think that autistic people see gender differently? - Have you noticed any impact of social difficulties/restricted and repetitive behaviours/sensory differences on the experience of gender dysphoria? |
| --- | --- |
|  | **Adaptations in services**   - Do you adapt your practice or work differently with autistic people with gender dysphoria compared to other patients in your service? - What have you found works, and what doesn’t work so well when supporting this group? - What are the barriers to supporting this group? - What information do clinicians need to work effectively with this group? |

**Appendix S3. Analytic Approach**

The process of IPA data analysis is described by Smith et al. (2009), and in this project we aimed to follow their guidance closely. The first step in analysis is *reading and re-reading* the transcripts in order to increase familiarity with important parts of the interview. The second step is *initial noting*, which involves a commitment to developing a detailed understanding of the transcript, and taking notes on what the participant is saying, and what this appears to mean to them. Smith et al. (2009) suggest using three overarching types of notes to structure the noting phase. The first of these are descriptive notes, which carefully describe the content or subject of the participants’ speech. The second are linguistic notes, which interpret the use of language by participants, for example use of metaphor or words to describe emotional experiences. Thirdly, conceptual notes move beyond the descriptive content to begin to synthesise this, and perhaps raise questions about the participants’ presentation of their experiences as found in the descriptive notes, and to consider underlying constructs which may be relevant. These interpretations must still be linked to the words spoken by the participant. This phase is traditionally done on paper on the margin of the transcript. The data for the young person and parent group were analysed in this way, either on paper or typed in a word document. For the adult study and the clinician groups, NVivo was used with the ‘annotations’ feature to conduct this part of the analysis. The third step is *developing emergent themes* and involves using the initial notes as the main source from which to develop initial themes from the transcript (Smith et al., 2009). Themes should summarise the notes taken in the second step, while maintaining a firmly phenomenological approach, that is, focusing on the meaning of the experience to the participant. This was conducted on the right hand margin of transcripts for the young person and parent study, and using the ‘nodes’ feature in Nvivo.

The fourth step is *searching for connections across emergent themes* which requires decision-making about how the themes might relate to one another, in order to highlight the central parts of the participant’s experience. Smith et al. (2009) suggest a number of techniques which can help in this process. This includes abstraction, or combining themes which relate to one another, or conversely, polarisation which involves placing emergent themes with opposite meanings together. Contextualisation involves paying attention to factors such as the narrative of participants, cultural themes, and temporal themes. Another technique is using function, that is, pulling together themes that served a particular purpose for the participant, for example, demonstrating their experience of overcoming adversity. On Nvivo this was done using the ‘nodes’ feature, and for the young person and parent study, this was done in excel to organise groups of themes together. The fifth step is *moving on to the next case,* and at this stage, findings from the previous transcripts should be bracketed, or set aside, in order to analyse the next transcript with a full focus on the lived experience of the next participant, which will be different to the previous participant. This ensures an idiographic approach throughout the project. The sixth, and final step is *looking for patterns across cases*, which can use similar strategies as employed in step four. This includes a commitment to maintaining the individual perspectives of participants, as well as looking for similarities across participants’ experiences.

This project moves beyond the traditional IPA approach to include a multiperspectival IPA approach (Larkin et al., 2019). It was hoped that understanding the intersection of autism and gender dysphoria from a range of perspectives would deepen the overall understanding of this phenomena and provide an account of the similarities and differences in perspectives between different groups. Larkin et al. propose some different types of multiperspectival IPA studies, and this study represents *directly related groups*, that is, distinct samples which have experience of the same phenomenon, but from different perspectives. All participants have been in close proximity to the experience of being autistic and experiencing gender dysphoria, either from personal experience, or from the outside through being a parent or clinician. In the case of clinicians, both groups were working with autistic people experiencing gender dysphoria, but one group worked with adults and the other with young people, giving them different perspectives. The children and young person stage of the analysis is an example of a family design, where parent-child dyads were interviewed to understand how young people and their parents experienced their gender identity and autism and accessing support for gender dysphoria.

Multiperspectival IPA proceeds with the analysis using the six steps above. One difference is that themes are pulled together within the units of study; in the case of the parent and child study, emergent themes were generated first for the young person, then their parent, and then for the unit of study: each dyad, before moving on to the next dyad. Similarly, in the clinician study, themes were generated at the individual participant level for each adult clinician first, before looking for patterns across all the adult clinician cases. Next, the same process was followed for the young person clinicians. When developing themes within a multiperspectival study, Larkin et al. (2019) suggest using a range of strategies. These include identifying consensus, when participants with different perspectives arrive at similar understandings, for example, both autistic adults and young people describing the distress due to a mismatch between their bodies and conflict, where participants disagree in their understandings, for example, parents seeing difficulties through the lens of autism, where young people see difficulties through the lens of gender. Another strategy is identifying complementary concepts, for example, clinicians expressing their struggles to adapt their communication with autistic patients, and autistic people feeling that clinicians do not always understand them. Finally, the work of Noblit and Hare (1988) inspires the strategy of finding *lines of argument* within the analysis, which involves drawing on the key themes identified to provide a coherent narrative which may share a structure with the system from which data was collected. A divergence from the original multiperspectival IPA design in this project was that the analysis was written for each individual group (adults; YP and parents; YP and adult clinicians), with finalised themes, and then these themes were re-analysed or synthesised in the final multiperspectival study. A typical multiperspectival IPA study would have fewer participants and so would generate themes for each group, which would then be described in one final analysis. Given the number of participants, with an in-depth, idiographic analytic approach maintained throughout data analysis, it was not feasible to present the findings in this way. Instead, we decided to produce three papers with preliminary findings, with a final synthesis paper with the overall multiperspectival analysis.

**Appendix S4. Reflexivity Statement**

The lead researcher is a cisgender woman, with no neurodevelopmental differences, and a clinical psychologist in the National Health Service (NHS), working with neurodiverse and gender diverse children and adolescents. Throughout this project the lead researcher was supported by a supervisory team of autism and gender experts, who are all clinical psychologists and academics who are cisgender and neurotypical. All three supervisors supervised the analysis of the data collected by the lead researcher. The supervisory team all had experience in supervising projects related to gender identity, with two of the supervisors having conducted research in gender identity clinics in the past. At the outset of the research, the team met to discuss their different experiences and viewpoints with regard to gender diversity and autism. Each team member explained their experience and preconceptions about the area, in order to practice reflexivity as a team, and to ensure that the findings of the research were as credible as possible. In order to maintain reflexivity throughout the project, the lead researcher kept a reflexive diary, attending IPA research group meetings, and discussed positionality regularly in IPA supervision meetings.
